# European thyroid journal: passing on the baton

**DOI:** 10.1530/ETJ-23-0216

**Published:** 2023-11-08

**Authors:** S H S Pearce, L Persani

**Affiliations:** 1 Translational and Clinical Research Institute, Faculty of Medical Sciences, Newcastle University, Newcastle upon Tyne, United Kingdom; 2 Department of Endocrine and Metabolic Diseases, IRCCS Istituto Auxologico Italiano, Milan, Italy

## Message from the outgoing editor-in-chief

*European Thyroid Journal* (*ETJ*) is the official journal of the European Thyroid Association (ETA) and has now been continuously published since 2012. The journal was the brainchild of Profs. Peter Laurberg and Wilmar Wiersinga, the later putting *ETJ* on a strong foundation as editor-in-chief until 2019. I was delighted to take on the editor-in-chief role in 2019, and with a 2022 impact factor of 4.7, it is now established in the second quartile of the Endocrinology and Metabolism category and as the top open-access journal completely dedicated to thyroid research. Changing our publisher to BioScientifica in 2021 along with embracing the fully open-access model with online publication has meant that the journal content is now fully and universally accessible to readers from any community. Furthermore, we can accept an unlimited number of papers each year, based purely on scientific quality, relevance and interest. I am delighted with the rise in manuscript submissions to* ETJ* and with the steady upward progress of the impact factor over the last 4 years. This reflects the faith of ETA members and numerous thyroid researchers from around the globe in sending their work to the journal, as well as the dedication and expertise of the editorial board and associate editors. I would like to take this opportunity to thank all the reviewers and members of the editorial board who have contributed to the journal over my time in the role of editor-in-chief. I am equally delighted to pass on the journal in what I believe is excellent health to Luca Persani as new editor-in-chief, who I know will do an excellent job in *ETJ*’s next phase of development.

SHS Pearce

## Message from the incoming editor-in-chief

It is a huge privilege for me to take on the responsibility of the official journal of the European Thyroid Association (ETA)!

We are acutely aware that authors within our field have an ever-increasing choice of open-access journals in which to submit their research manuscripts. However, we believe that the international reputation for quality and scientific rigor of our journal, being the official organ of ETA, together with continued hard work from the editorial team and our publishing colleagues, will help us in our aim of maintaining *ETJ* as the preferred choice of authors for their valued manuscripts. In return, we offer them a rapid decision from submission to first response. I believe that primary screening should become more rigorous as the most critical and fair step in selecting papers with the required novelty and attraction; the reviewing process should then become a secondary step available for a limited number of the submitted papers. External reviewers will be asked to provide fair, constructive and informative feedback and authors should feel the reviewing process is a necessary step aimed at further improving the original manuscripts. With a dedicated and enlarged group of associate editors and editorial board (EB) members this will be possible so that I believe we shall become more and more competitive with several other journals publishing thyroid research. We all wish to see *ETJ* becoming the voice of world leading scientists in the thyroid field. We would like to enlarge further the visibility of *ETJ* by involving in the EB highly motivated, clinical and basic, scientists coming also from under-represented areas of the world and different sub-specialties in thyroid field.

Being an online open-access journal, *ETJ* can expand the number of illustrative items included in a paper. From the start of the new editorial tenure, we will encourage authors of accepted manuscripts to prepare illustrative graphical abstracts of their findings, which may also be useful for congress presentations or teaching, but also showcasing the work on social media. We would like to receive research papers with nice and informative illustrations of their findings aimed to attract the *ETJ* readers. We also would like to receive new proposals for short or extensive reviews on hot topics in thyroid research. Reviews should be authoritative, signed by a limited number of authors with a documented expertise on the specific topic; this is unfortunately not always the case in several journals.

So, we look forward, with optimism, to producing a high-quality journal, which captures the most exciting and novel breakthroughs in thyroid basic, translational and clinical science.

L Persani

## Declaration of interest

The authors declare that there is no conflict of interest that could be perceived as prejudicing the impartiality of this editorial.

## Funding

The authors did not receive any specific grant from any funding agency in the public, commercial or not-for-profit sector.

